# A new stage for predicting the prognosis of granulomatous lobular mastitis

**DOI:** 10.1371/journal.pone.0319956

**Published:** 2025-03-19

**Authors:** Ruiyang Wu, Haiyan Zhang, Yan Wang, Yunlu Mo, Huihua Hu, Jin Chen, Wei Huang, Qinyan Shi, Yuqing Kang, Jing Luo

**Affiliations:** 1 Department of Breast and Thyroid, Sichuan Provincial Hospital for Women and Children (Affiliated Women and Children’s Hospital of Chengdu Medical College), Chengdu, China; 2 Department of Pathology, Sichuan Provincial Hospital for Women and Children (Affiliated Women and Children’s Hospital of Chengdu Medical College), Chengdu, China; Children's National Hospital, George Washington University, UNITED STATES OF AMERICA

## Abstract

**Introduction:**

The prognosis of granulomatous lobular mastitis (GLM) had been poor, primarily due to the lack of a unified disease assessment standard.

**Objective:**

The purpose of this cohort study was to establish a staging system for GLM to more accurately evaluate the prognosis of patients.

**Methods:**

This study retrospectively collected data from 264 GLM patients who visited our hospital between January 2017 and December 2023. Through logistic regression analysis, factors associated with prognosis were identified, which served as the basis for creating a new staging system.

**Results:**

Univariate and multivariate logistic regression analysis revealed that hyperlipidemia (HR: 2.031; 95% CI: 1.100-3.750) and microabscesses (HR: 2.087; 95% CI = 1.138-3.827) were significant independent risk factors affecting the prognosis of GLM patients. Based on the results of logistic analysis, three different stages were ultimately established, and it was found that stage C had the highest AUC value (AUC: 0.642), followed by stage B (AUC: 0.628), with stage A (AUC: 0.614) having the lowest. The Delong test revealed no significant difference in AUC values between stage A and stage B (P =  0.255), nor between stage B and stage C (P =  0.263). However, the AUC value of the stage C was found to be higher than that of stage A (P <  0.001). Given that stage C has the highest AUC value, this study selected stage C as the final stage for evaluating the prognosis of GLM patients and named it the 1st edition of GLM stage.

**Conclusions:**

This study constructed a rigorous and widely applicable GLM staging system (the 1st edition of GLM stage). The system demonstrated good predictive outcomes and provided strong support for clinical decision-making.

## Introduction

Granulomatous lobular mastitis (GLM) was a chronic benign inflammatory disease of the breast that occurred in the lobules [1]. In recent years, there has been a marked increase in the incidence of GLM [2], with a particularly notable uptrend observed in the Mediterranean region and among developing countries in Asia, including China [3].

Despite being a benign condition, GLM, there were several pieces of evidence to support that it can severely impact patients’ health and quality of life. Firstly, pain, breast swelling, breast lumps, and inflammatory changes akin to breast abscesses were the typical initial presentations of the disease [4]; yet, as the disease progressed, the local area frequently developed ulcers, scars, and a retraction of the skin and nipples, and even the entire breast might have shrunk, with atrophy potentially being severe, leading to the loss of up to 80% of mammary tissue [5]. Moreover, inflammation was a normal immune response to infections and tissue damage that sustained host defense and homeostasis [6], but chronic or dysregulated inflammation drove tumor initiation, progression, and metastasis by fostering an immunosuppressive tumor microenvironment and activating oncogenic signaling pathways [7–9], with mastitis history notably increasing cancer risk [10–12]. More significantly, the entire disease course lasted 6-12 months [13,14], with a recurrence rate as high as 10%-20% [15–19]. It became clear that, if not promptly managed, the disease would not only have inflicted damage upon the breast but also would have exacted a toll on the patient’s overall health and well-being, including their economic stability.

GLM was also referred to as the “incurable cancer”. Owing to its clinical and radiological characteristics resembling those of breast cancer [1], coupled with its inclination to relapse, it was frequently misidentified as breast cancer in the past [20]. Indeed, it bore a striking resemblance to breast cancer to a certain degree. Firstly, breast cancer was caused by the invasion of surrounding tissues by cancer cells, while GLM was thought to be caused by corynebacterium infection in breast tissue [1,21,22]. Additionally, chemotherapeutic agents exerted a lethal effect on cancer cells, whereas lipophilic antibiotics demonstrated efficacy in treating breast infections associated with corynebacterium species [23]. Ultimately, both conditions were capable of recurrence, with the distinction that GLM recurred solely within the breast tissue, whereas breast cancer had the potential to metastasize to distant sites.

Nevertheless, the prognosis of GLM was entirely distinct from that of breast cancer. The recurrence rate of breast cancer was less than 1% [24], while the recurrence rate of GLM was as high as 10%-20% [15–19]. This significant difference underscored the unique challenges posed by GLM in terms of management and patient outcomes. Besides its unclear pathogenesis, significant individual variability, and lack of standardized treatment approaches, the primary reason for the challenges in managing GLM was the absence of a unified standard for disease assessment. This lack of standardization could lead to confusion and misdiagnosis, as the criteria for evaluating the severity and progression of GLM were not well-established, unlike breast cancer, which had more defined assessment standards. Consequently, this study, taking the staging system of breast cancer as a reference, has preliminarily established a staging system for GLM to more accurately assess patient prognosis.

## Materials and methods

### Data and samples

The data used in this retrospective study originated from a private database established by the department of breast and thyroid of our hospital in March 2022, and named the Non-Puerperal Mastitis Database (NPMD). The database retrospectively collected data on patients with non-lactating mastitis who visited Sichuan Provincial Hospital for Women and Children starting from January 2017 and conducted regular follow-ups. On July 1, 2024, this study accessed the database and collected the medical data of 533 patients diagnosed with GLM at Sichuan Provincial Hospital for Women and Children from January 2017 to December 2023. Since the primary endpoint of this study was the 1-year cure rate, patients who had follow-up periods of less than one year and had not achieved cure were excluded from the analysis. Ultimately, this study enrolled a total of 264 patients.

Cure time was calculated in months from the initial consultation date to the date of cure or the last follow-up. The last follow-up occurred in June 2024. In this study, the endpoint event was cure, which was defined by the complete absence of symptoms, the disappearance of the lump, and negative findings on physical and imaging assessments [25]. The median duration of follow-up was 15 months (ranging from 1 to 72 months), and the one-year cure rate was 72.7%.

The study included factors such as age at diagnosis, body mass index, origin of primary, the diameter of the largest lesion visible under ultrasound, the number of lesions visible under ultrasound, white blood cell, nipple inversion, and so on. Among them, antinuclear antibody profile encompassed 15 distinct markers (anti-mitochondrial autoantibodies M2, ribosomal protein P0, double-stranded DNA, proliferative cell nuclear antigen, centromere protein B, anti-Jo-1 antibodies, polymyositis/scleroderma, scleroderma-associated antigen, sjögren’s syndrome B, anti-Ro/SSA-52kD antibodies, anti-Ro/SSA-60kD antibodies, smith antigen, and uuclear ribonucleoprotein/smith antigen), with the presence of a positive result in any single marker being indicative of a positive antinuclear antibody profile. The diagnostic criteria for hyperlipidemia were that one of the following conditions in the blood must be met: total cholesterol of 220 mg/dL or above, low-density lipoprotein cholesterol (LDL-C) of 140 mg/dL or above, high-density lipoprotein cholesterol below 40 mg/dL, and triglycerides of 150 mg/dL or above [26]. Some of the data in this study were missing. Using the mice package in R for multiple imputation (MI) has addressed this issue, with a seed of 1 and the imputation method being random forest [27].

### Statistical analysis

Continuous variables were expressed as means and standard deviations and were compared using a T-test. Categorical variables were compared using the Chi-square test and were expressed as frequencies and percentages. Through logistic regression analysis, we identified several independent prognostic factors that were significantly associated with the outcome of interest. These factors were carefully examined and their predictive value was assessed to ensure they were robust indicators of prognosis. Once these factors were established, they were used as the basis for creating a new staging system. When a continuous variable was used as a staging criterion, the X-tile software was employed to obtain the optimal cutoff value for that continuous variable [28]. The X-tile software was a valuable tool used by researchers and clinicians to determine optimal cutoff points for continuous variables in medical and biological research. It allowed for the analysis of data to classify patients into different risk groups, which is particularly useful in the development of staging systems for diseases. The predictive power of staging was assessed using the area under the curve (AUC) value, which is derived from the Receiver Operating Characteristic (ROC) curve. The higher the AUC value, the better the predictive ability of the staging system. All hypothesis tests were two-tailed, and P <  0.05 was considered statistically significant. Statistical analysis were conducted using R software 4.2.2.

The private database complied with the Declaration of Helsinki (revised in 2013) and had been approved by the Medical Ethics Committee of Sichuan Provincial Hospital for Women and Children (approval number: 20220330-024). Since the database only retrospectively collected patient data and did not involve any intervention measures for patients, and the data was anonymized, the requirement for informed consent had been waived.

## Results

### Descriptive characteristics

The study included a cohort of 264 patients diagnosed with GLM, all of whom were female. The dataset encountered missing values for various parameters, such as the diameter of the largest lesion visible under ultrasound (7.5% were missing), the number of lesions visible under ultrasound (7.9% were missing), white blood cells (14.3% were missing), anti-Streptolysin O (with a missing rate of 60.2%), rheumatoid factor (60.6% were missing), C-reactive protein (53.0% were missing), and antinuclear antibody profile (60.2% were missing), among others. Table 1 displayed the comparison of demographic and pathological characteristics between the two groups of patients before and after MI. The statistical analysis of the baseline characteristics indicated that the p-values from the comparisons conducted before and after MI were all greater than 0.05. This suggested that the imputation method did not introduce additional bias into the analysis. Therefore, it was concluded that there were no statistically significant differences in the baseline characteristics between the two groups of patients, whether the data was assessed before or after the application of MI.

**Table 1:** The demographic and pathological characteristics of GLM patients before and after MI.

Factors	Data integrity, N(%)	Before MI	After MI	P value
		N(%)/x ± s	N(%)/x ± s	
Body mass index	262(99.3)	23.9 ± 3.9	23.9 ± 3.9	0.918
The diameter of the largest lesion visible under ultrasound (mm)	244(92.5)	43.4 ± 20.7	43.2 ± 20.6	0.897
The number of lesions visible under ultrasound	243(92.1)	4.6 ± 2.9	4.5 ± 2.8	0.706
White blood cell (10^9/L)	226(85.7)	9.9 ± 3.9	9.8 ± 3.8	0.658
Anti-Streptolysin O (IU/ml)	105(39.8)	66.8 ± 70.6	69.0 ± 78.5	0.799
Rheumatoid factor (IU/ml)	104(39.4)	6.9 ± 7.8	6.9 ± 6.2	0.998
C-reactive protein (mg/L)	124(47.0)	18.5 ± 23.5	17.6 ± 23.0	0.734
Antinuclear antibody profile	105(39.8)			0.221
negative		90(85.7)	238(90.2)	
positive		15(14.3)	26(9.8)	
Immunoglobulin G (g/L)	106(40.2)	12.6 ± 2.4	12.5 ± 2.4	0.784
Immunoglobulin M (g/L)	106(40.2)	1.6 ± 0.7	1.7 ± 0.7	0.600
Immunoglobulin A (g/L)	106(40.2)	2.4 ± 0.9	2.5 ± 0.9	0.731
Immunoglobulin E (g/L)	106(40.2)	80.7 ± 107.2	69.6 ± 91.6	0.315
Complement C3 (g/L)	106(40.2)	1.3 ± 0.3	1.3 ± 0.3	0.451
Complement C4 (g/L)	106(40.2)	0.4 ± 0.1	0.4 ± 0.1	0.193
Prolactin (uIU/ml)	220(83.4)	695.4 ± 963.2	704.9 ± 1008.8	0.916
Hyperlipidemia	142(53.8)			0.959
no		76(53.5)	142(53.8)	
yes		66(46.5)	122(46.2)	
Microabscess	186(70.5)			0.721
no		87(46.8)	128(48.5)	
yes		99(53.2)	136(51.5)	

### Prognostic factor analysis

Univariate and multivariate logistic regression analyses were conducted to evaluate the factors associated with the prognosis of patients with GLM. The findings revealed that hyperlipidemia (HR: 2.031; 95% CI: 1.100-3.750; P =  0.024; Table 2) and microabscess (HR: 2.087; 95% CI: 1.138-3.827; P =  0.017; Table 2) emerged as independent risk factors significantly impacting the prognosis of GLM patients.

**Table 2:** Univariate and multivariate analysis of prognostic factors for 264 patients with GLM.

Factors	N(%)/x ± s	Univariate	Multivariate	
		P value	HR(95%CI)	P value
Age at diagnosis (years)	31.4 ± 5.0	0.847	1.002(0.944-1.065)	0.940
Body mass index	23.9 ± 3.9	0.382	1.028(0.954-1.109)	0.468
Origin of primary		0.531		0.519
left	147(55.7)		Ref	
right	115(43.6)		0.701(0.382-1.288)	
bilateral	2(0.8)		0.885(0.036-21.978)	
The diameter of the largest lesion visible under ultrasound (mm)	43.2 ± 20.6	0.105	1.009(0.994-1.024)	0.256
The number of lesions visible under ultrasound	4.5 ± 2.8	0.175	1.064(0.945-1.198)	0.305
White blood cell (10^9/L)	9.8 ± 3.8	0.772	1.001(0.918-1.091)	0.985
Anti-Streptolysin O (IU/ml)	69.0 ± 78.5	0.985	1.000(0.996-1.004)	0.867
Rheumatoid factor (IU/ml)	6.9 ± 6.2	0.342	1.031(0.983-1.082)	0.205
C-reactive protein (mg/L)	17.6 ± 23.0	0.555	0.994(0.979-1.009)	0.403
Antinuclear antibody profile		0.070		0.038
negative	238(90.2)		Ref	
positive	26(9.8)		0.241(0.063-0.923)	
Immunoglobulin G (g/L)	12.5 ± 2.4	0.866	1.028(0.902-1.171)	0.678
Immunoglobulin M (g/L)	1.7 ± 0.7	0.517	1.311(0.843-2.038)	0.230
Immunoglobulin A (g/L)	2.5 ± 0.9	0.491	0.815(0.575-1.155)	0.250
Immunoglobulin E (g/L)	69.6 ± 91.6	0.451	0.998(0.995-1.002)	0.364
Complement C3 (g/L)	1.3 ± 0.3	0.775	0.798(0.204-3.114)	0.745
Complement C4 (g/L)	0.4 ± 0.1	0.645	0.299(0.015-5.821)	0.425
Prolactin (uIU/ml)	704.9 ± 1008.8	0.054	1.000(1.000-1.001)	0.022
Hyperlipidemia		0.033		0.024
no	142(53.8)		Ref	
yes	122(46.2)		2.031(1.100-3.750)	
Mammary abscess		0.162		0.075
no	39(14.8)		Ref	
yes	225(85.2)		2.408(0.915-6.335)	
Microabscess		0.030		0.017
no	128(48.5)		Ref	
yes	136(51.5)		2.087(1.138-3.827)	
Nipple inversion		0.428		0.662
no	157(59.5)		Ref	
yes	107(40.5)		1.146(0.622-2.111)	

### Initial stage

Based on the outcomes of univariate and multivariate prognostic analyses, patients were stratified into four distinct groups: Group a, comprising patients without hyperlipidemia or microabscess; Group b, consisting of patients with hyperlipidemia but devoid of microabscess; Group c, including patients with microabscess without hyperlipidemia; and Group d, encompassing patients with both hyperlipidemia and microabscess. A chi-square test was utilized to assess the 1-year cure rates across these groups, revealing that Group d exhibited a markedly lower 1-year cure rate (57.6%) than the other groups (all P <  0.05, Table 3). In contrast, no statistically significant differences in the 1-year cure rates were observed among Groups a, b, and c.

**Table 3:** Comparison of 1-year cure rates among group a, b, c, and d (N = 264).

Group	N (%)	1-year cure rate (%)	P value	P value	P value
a	65(24.6)	83.1	Ref		
b	63(23.9)	74.6	0.240	Ref	
c	77(29.2)	74.0	0.193	0.938	Ref
d	59(22.3)	57.6	0.002	0.047	0.044

Due to the similarity and lack of statistical difference in the 1-year cure rates between group b and group c, they were combined into a single group bc. It was observed that group a had the highest cure rate, followed by the group bc, with group d having the lowest (Table 4). A chi-square test revealed that 1-year cure rate of group d (57.6%) was lower than that of group a (83.1%, P =  0.002) and bc (74.3%, P =  0.020), while there was no statistically significant difference in the 1-year cure rates between group a and bc (P =  0.164, Table 4).

**Table 4:** Comparison of 1-year cure rates among group a, bc, and d (N = 264).

Group	N (%)	Definition	1-year cure rate (%)	P value	P value
a	65(24.6)	without hyperlipidemia or microabscess	83.1	Ref	
bc	140(53.0)	with either hyperlipidemia or microabscess	74.3	0.164	Ref
d	59(22.3)	with both hyperlipidemia and microabscess	57.6	0.002	0.020

### Subgroup analysis

To enhance the stratification of prognostic outcomes among patients in group a, group bc, and group d, a series of univariate and multivariate logistic regression analyses were performed within each group to determine the factors influencing their prognoses. In the case of group a patients, no independent prognostic factors were identified that could predict the 1-year cure rate (Table 5). However, for group bc patients, the univariate logistic regression revealed that prolactin significantly impacted the 1-year cure rate (P =  0.038, Table 5). Subsequent multivariate analysis in this group identified mammary abscess as a significant correlate of the 1-year cure rate (P =  0.046, Table 5). Lastly, within group d, the diameter of the largest lesion visible under ultrasound emerged as a critical independent prognostic factor (P =  0.029, Table 5).

**Table 5:** Univariate and multivariate analysis of prognostic factors in group a, bc, and d.

Factors	Group a (N = 65)		Group bc (N = 140)		Group d (N = 59)	
	Univariate	Multivariate	Univariate	Multivariate	Univariate	Multivariate
	P value	P value	P value	P value	P value	P value
Age at diagnosis (years)	0.405	0.892	0.792	0.455	0.814	0.896
Body mass index	0.350	0.939	0.425	0.311	0.887	0.671
Origin of primary	0.122	0.091	0.877	0.955	0.992	0.988
The diameter of the largest lesion visible under ultrasound (mm)	0.743	0.216	0.600	0.715	0.046	0.029
The number of lesions visible under ultrasound	0.179	0.188	0.657	0.457	0.734	0.528
White blood cell (10^9/L)	0.982	0.167	0.579	0.390	0.784	0.364
Anti-Streptolysin O (IU/ml)	0.064	0.391	0.582	0.369	0.750	0.800
Rheumatoid factor (IU/ml)	0.506	0.122	0.860	0.972	0.580	0.073
C-reactive protein (mg/L)	0.261	0.094	0.315	0.180	0.292	0.203
Antinuclear antibody profile	0.999	0.998	0.459	0.626	0.209	0.339
Immunoglobulin G (g/L)	0.775	0.165	0.940	0.975	0.607	0.858
Immunoglobulin M (g/L)	0.526	0.310	0.534	0.174	0.740	0.345
Immunoglobulin A (g/L)	0.459	0.078	0.334	0.883	0.130	0.159
Immunoglobulin E (g/L)	0.271	0.247	0.934	0.697	0.602	0.481
Complement C3 (g/L)	0.937	0.383	0.451	0.963	0.323	0.422
Complement C4 (g/L)	0.076	0.333	0.371	0.855	0.642	0.947
Prolactin (uIU/ml)	0.536	0.355	0.038	0.050	0.421	0.307
Mammary abscess	0.520	0.634	0.069	0.046	0.553	0.231
Nipple inversion	0.542	0.521	0.645	0.832	0.767	0.479

Based on the aforementioned findings, we have chosen prolactin, mammary abscess, and the diameter of the largest lesion visible under ultrasound as the stratification factors for further subgroup analysis. Recognizing that prolactin and the diameter of the largest lesion visible under ultrasound are continuous variables, we employed X-tile software to ascertain the most discriminating cut-off points for these variables to enhance the subgroup analysis. The analysis conducted by the software indicated that the optimal threshold values for prolactin and the diameter of the largest lesion visible under ultrasound are 697 (uIU/ml) and 39 (mm), respectively.

### Final stage

Based on the outcomes of the univariate and multivariate logistic analyses from three patient groups, we selected prolactin, mammary abscess, and the diameter of the largest lesion visible under ultrasound as the criteria for further stratification. Consequently, the group bc was divided into group e ( < 700 uIU/ml) and group f ( ≥ 700 uIU/ml) based on prolactin levels. Additionally, the group bc was categorized into group g (without mammary abscess) and group h (with mammary abscess) according to the presence of mammary abscess. Furthermore, group d was stratified into group i ( < 40 mm) and group j ( ≥ 40 mm) based on the diameter of the largest lesion visible under ultrasound. These classifications culminated in the establishment of three distinct stages (Table 6): Stage A, Stage B, and Stage C.

**Table 6:** The content of three distinct stages.

Stage A			Stage B			Stage C		
Group	N	one-year cure rate (%)	Group	N	one-year cure rate (%)	Group	N	one-year cure rate (%)
a	65	83.1	a	65	83.1	a	65	83.1
bc	140	74.3	e	108	75.0	g	22	90.9
			f	32	71.9	h	118	71.2
d	59	57.6	i	25	76.0	i	25	76.0
			j	34	44.1	j	34	44.1

**Fig 1:**
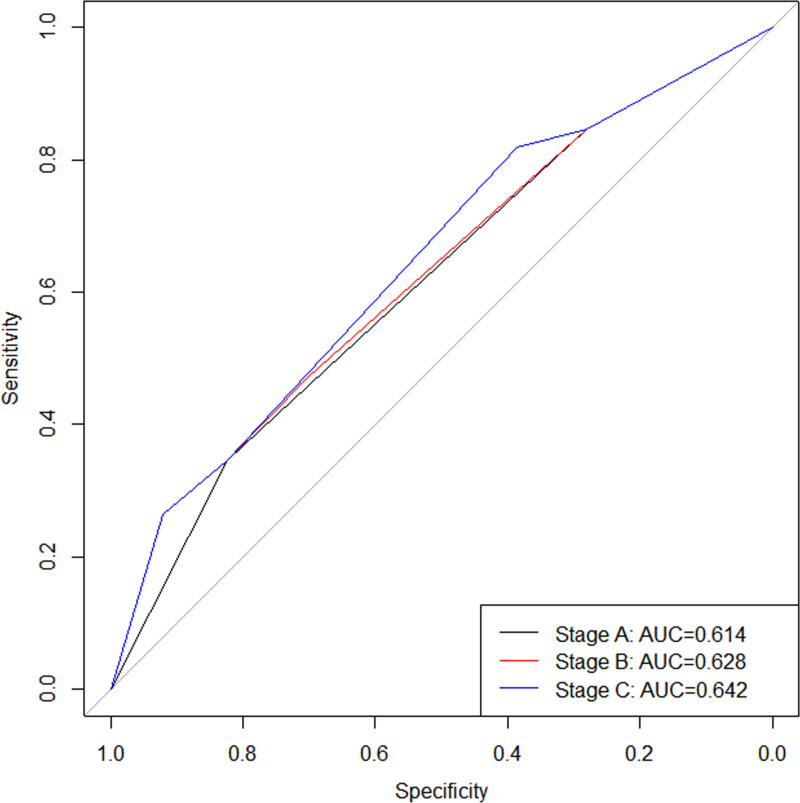
ROC curves for stages A, B, and C.

We selected the AUC value to evaluate the predictive performance of the three stages and found that stage C had the highest AUC value (AUC: 0.642; 95%CI: 0.571-0.714; Fig 1), followed by stage B (AUC: 0.628; 95%CI: 0.553-0.702; Fig 1), with stage A (AUC: 0.614; 95%CI: 0.544-0.684; Fig 1) having the lowest. The Delong test revealed no significant difference in AUC values between stage A and stage B (P =  0.255), nor between stage B and stage C (P =  0.263, Fig 1). However, the AUC value of the stage C was found to be higher than that of stage A (P <  0.001, Fig 1). Given that stage C has the highest AUC value (Fig 1), this study selected stage C as the final stage for evaluating the prognosis of GLM patients and named it the 1st edition of GLM stage. The specific staging details can be found in Table 7.

**Table 7:** The content of the 1st edition of GLM stage.

Stage	Content
I	During the entire course of the disease, the patient had neither hyperlipidemia nor microabscess.
II	During the entire course of the disease, the patient had either hyperlipidemia or microabscess.
a	There was no formation of mammary abscess throughout the entire course of the disease.
b	Mammary abscess formation occurred throughout the entire course of the disease.
III	During the entire course of the disease, the patient had both hyperlipidemia and microabscess.
a	The diameter of the largest lesion visible under ultrasound was less than 4 cm.
b	The diameter of the largest lesion visible under ultrasound was 4 cm or greater.

## Discussion

Although GLM was a benign condition, its poor local manifestations had led to redness, swelling, ulceration, and even deformity of the breast, severely affecting the health and quality of life of patients. The reason for the poor prognosis may have been the lack of a consistent standard for disease assessment and the non-uniformity in treatment methods. To address this situation, we referred to breast cancer to establish a staging system for GLM to assess patient prognosis. This study employed logistic analysis to pinpoint the factors influencing patient prognosis and subsequently restructured these factors into a comprehensive staging system.

This study identified hyperlipidemia as an independent prognostic factor (HR: 2.031; 95% CI: 1.100-3.750), suggesting that patients with hyperlipidemia were at over twice the risk of experiencing a worse prognosis compared to those without hyperlipidemia. So far, little was known about the relationship between GLM and lipid metabolism, and to our knowledge, this was the first time it was reported that hyperlipidemia might affect the prognosis of GLM. However, the following reasons may have supported the results of this study. Initially, corynebacterium species, especially C. kroppenstedtii, were considered to be the main etiological agents of GLM [1,21,22], with C. kroppenstedtii being a fastidious lipophilic bacterium whose growth was dependent on a lipid-rich culture medium [29–31]. Secondly, current research has indicated that lipophilic antibiotics may have offered superior efficacy in treating breast infections associated with Corynebacterium species [23]. Furthermore, reports have indicated that obesity was linked to low-grade chronic inflammation [32], potentially undermining the local immune defenses within the mammary glands [33]. Lastly, a study has revealed that, in contrast to the group with benign breast masses, individuals with non-puerperal mastitis exhibited decreased levels of high-density lipoprotein cholesterol [34]. Within the non-puerperal mastitis cohort, patients with GLM demonstrated markedly elevated LDL-C levels in comparison to those with periductal mastitis [34]. In summary, these findings underscores the importance for both physicians and patients to prioritize the management of hyperlipidemia, as it may exert a significant influence on the treatment efficacy and recovery of individuals with GLM.

The pathological manifestations of GLM were primarily centered around lobular non-caseating granulomatous inflammatory reactions and chronic inflammatory responses, often accompanied by microabscesses. Microabscesses were a distinctive pathological feature of GLM, with the abscess cavity primarily composed of neutrophils, along with macrophages and necrotic tissue, surrounded by epithelioid cells and mononuclear cells [35,36]. In this study, approximately half of the patients’ tissue specimens contained microabscesses, and microabscesses were identified as an independent prognostic factor with a higher hazard ratio (HR: 2.087). This meant that the presence of microabscesses significantly increased the risk of adverse outcomes, specifically, patients with microabscesses were more than twice as likely to have poor outcomes compared to those without microabscesses. Despite the limited research that had been available on the relationship between microabscesses and the prognosis of patients with GLM, several possible reasons could have supported the findings of this study. ‌Firstly‌, in the surgical treatment of GLM, microabscesses were difficult to identify with the naked eye, which could have led to incomplete resection of the lesion during surgery, thereby increasing the risk of recurrence. Moreover, under the microscope, it is common to see epithelioid cells surrounding the abscess cavity, coupled with edema and fibrosis in the surrounding tissue. Even with incision and drainage, the results may be poor, increasing the difficulty of treatment. ‌Lastly‌, the bacteria and necrotic tissue within the abscess cavity also complicated the local inflammation, which may have led to further damage to breast tissue, and even resulted in long-term damage, such as glandular atrophy and loss of function, increasing the difficulty of treatment and ultimately leading to the chronicity of the disease. In summary, this indicated that microabscesses played a significant role in the treatment and prognosis evaluation of GLM. Their presence may have suggested a higher risk of recurrence and a more complex treatment process. Therefore, in clinical practice, the identification and management of microabscesses may have been of great importance for improving patient outcomes.

This study discovered that the diameter of the largest lesion visible under ultrasound might affect the prognosis of GLM patients, a result that was to some extent consistent with the findings of other studies [37]. For instance, Kaviani et al. [37] classified the severity of GLM based on lesion diameter and symptoms, where mild disease was characterized by a breast mass of less than 2 cm, moderate disease by a mass between 2-5 cm, and severe disease by a breast mass greater than 5 cm. Concurrently, they observed that in mild to moderate cases, the condition exhibited a self-limiting nature [37]. Theoretically, a larger lesion diameter implied a more extensive disease focus, which was associated with a higher likelihood of recurrence. This concept was supported by various studies that indicated a correlation between lesion diameter and clinical outcomes such as recurrence and metastasis [38]. In the context of GLM, a larger diameter might have suggested a more widespread disease presence within the breast tissue, potentially leading to a higher risk of recurrence due to the possibility of more extensive involvement of surrounding tissues and a greater number of cells harboring the disease. This theoretical perspective was aligned with the understanding that larger lesions may have had a more complex interaction with the immune system and the microenvironment, which could have influenced disease progression and response to treatment. Therefore, based on these results, we concluded there might have been a correlation between lesion diameter and the condition of GLM.

The research revealed that the formation of abscesses could potentially have a significant impact on the prognosis of patients with GLM. Abscess formation was a common complication of GLM, which could have led to skin ulceration and even caused breast deformities. Multiple studies had demonstrated that the presence of abscesses increased the recurrence rate in GLM patients [39–41], but the specific reasons were still unclear. A study utilizing 16S ribosomal DNA sequencing found that Corynebacterium species was the sole genus present in the pus of GLM patients, showing a significant difference from other groups [42]. Furthermore, research indicated that individuals with GLM who were afflicted by Corynebacterium infections were confronted with a recurrence risk that was 2.64 times elevated [43]. This could be one of the contributing factors explaining why GLM patients with abscess formation were particularly susceptible to recurrence.

This study identified factors affecting patient prognosis using logistic regression analysis. After several rounds of re-analysis, a relatively standardized staging system was developed, which demonstrated good predictive efficiency and could assist in clinical decision-making. To our knowledge, due to the limited number of GLM patient samples and the highly complex and diverse nature of the disease, other studies have only been able to conduct preliminary classifications based on patient symptoms [37,44,45]. This simplified classification method has limited the possibility of a deeper understanding of the disease and has been detrimental to the conduct of further scientific research and the development of refined treatment strategies. For instance, in the international multidisciplinary consensus published in 2021 [44], GLM was classified into self-limited stage, congestive swelling stage, abscess formation stage, and complex refractory stage based on tumor size and clinical symptoms. Kaviani et al. [37] similarly chose a very simple method to grade the severity of GLM, arbitrarily dividing it into three broad categories: mild, moderate, and severe. These classification methods lacked the complexity and rigor of a standardized scoring system. Furthermore, two studies have evaluated the recurrence risk of GLM [46,47]. Yılmaz et al. [46] had developed a scoring system for granulomatous mastitis and had considered it an effective tool for predicting the risk of recurrence. However, their study had only included 53 samples and had not provided detailed data on the predictive capabilities of the scoring system, such as AUC values, which are key indicators. Another study [47] particularly emphasized the serum albumin-globulin ratio as an independent risk factor for predicting the recurrence risk of GLM and provided its predictive efficacy with an AUC value. Despite this study offering a quantitative indicator for assessing recurrence risk, it did not further develop a comprehensive staging system, and the limitation of sample size somewhat diminished its application potential and universality in clinical decision-making. To put it differently, as of the information available, a mature system for classifying the stages of GLM was absent, posing challenges for medical professionals in terms of prognostic stratification of their patients. However, there were several reasons that demonstrated the precision and broad applicability of the staging method provided by this study. First and foremost, our extensive sample size served as a cornerstone for the robustness and reliability of our research outcomes. Subsequently, we employed AUC metrics to meticulously evaluate our staging system, thereby substantiating the precision of its evaluative capabilities. Following that, we streamlined the staging process, reducing its complexity and enhancing its applicability across a variety of contexts. In addition, with the passage of time, our database had witnessed a steady annual expansion in the volume of samples. In the future, we will continue to refine our staging system to more accurately assess patients’ prognoses and to select the most appropriate treatment options for them.

Although our study introduced a relatively standardized staging system, there were several limitations that warrant acknowledgment. Firstly, the retrospective nature of the study necessitated the exclusion of some patients due to insufficient follow-up, which consequently reduced the number of patients included in the analysis. Secondly, the impact of treatment regimens on patient outcomes, a critical factor, was not incorporated into our staging system. Thirdly, the AUC value of our staging system was below the desired threshold of 0.7, indicating significant room for improvement in predictive accuracy. Lastly, due to the constraints of sample size, the model we developed was only internally validated, lacking the external validation that would confirm its generalizability. We had hoped that future prospective multicenter studies would address and overcome the limitations encountered in our previous research. By adopting a forward-looking and collaborative approach, these studies were expected to provide more robust and generalizable findings, thereby enhancing the reliability and applicability of the staging system.

## Conclusions

This study revealed that hyperlipidemia and microabscesses were independent prognostic factors. Based on these findings, we constructed a rigorous and widely applicable GLM staging system. The system demonstrated good predictive outcomes and provided strong support for clinical decision-making.
